# Complementary methods for structural assignment of isomeric candidate structures in non-target liquid chromatography ion mobility high-resolution mass spectrometric analysis

**DOI:** 10.1007/s00216-023-04852-y

**Published:** 2023-07-15

**Authors:** Masoumeh Akhlaqi, Wei-Chieh Wang, Claudia Möckel, Anneli Kruve

**Affiliations:** 1 Department of Materials and Environmental Chemistry, Svante Arrhenius väg 16C, 114 18 Stockholm, Sweden; 2 Department of Environmental Science, Svante Arrhenius väg 8, 114 18 Stockholm, Sweden

**Keywords:** Water analysis, Non-targeted analysis, Machine learning, Cyclic IMS, Liquid chromatography, High-resolution mass spectrometry

## Abstract

Non-target screening with LC/IMS/HRMS is increasingly employed for detecting and identifying the structure of potentially hazardous chemicals in the environment and food. Structural assignment relies on a combination of multidimensional instrumental methods and computational methods. The candidate structures are often isomeric, and unfortunately, assigning the correct structure among a number of isomeric candidate structures still is a key challenge both instrumentally and computationally. While practicing non-target screening, it is usually impossible to evaluate separately the limitations arising from (1) the inability of LC/IMS/HRMS to resolve the isomeric candidate structures and (2) the uncertainty of in silico methods in predicting the analytical information of isomeric candidate structures due to the lack of analytical standards for all candidate structures. Here we evaluate the feasibility of structural assignment of isomeric candidate structures based on in silico–predicted retention time and database collision cross-section (*CCS*) values as well as based on matching the empirical analytical properties of the detected feature with those of the analytical standards. For this, we investigated 14 candidate structures corresponding to five features detected with LC/HRMS in a spiked surface water sample. Considering the predicted retention times and database *CCS* values with the accompanying uncertainty, only one of the isomeric candidate structures could be deemed as unlikely; therefore, the annotation of the LC/IMS/HRMS features remained ambiguous. To further investigate if unequivocal annotation is possible via analytical standards, the reversed-phase LC retention times and low- and high-resolution ion mobility spectrometry separation, as well as high-resolution MS^2^ spectra of analytical standards were studied. Reversed-phase LC separated the highest number of candidate structures while low-resolution ion mobility and high-resolution MS^2^ spectra provided little means for pinpointing the correct structure among the isomeric candidate structures even if analytical standards were available for comparison. Furthermore, the question arises which prediction accuracy is required from the in silico methods to par the analytical separation. Based on the experimental data of the isomeric candidate structures studied here and previously published in the literature (516 retention time and 569 *CCS* values), we estimate that to reduce the candidate list by 95% of the structures, the confidence interval of the predicted retention times would need to decrease to below 0.05 min for a 15-min gradient while that of *CCS* values would need to decrease to 0.15%. Hereby, we set a clear goal to the in silico methods for retention time and *CCS* prediction.

**Graphical abstract:**

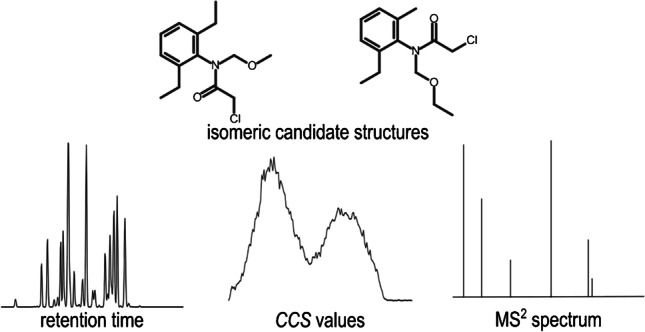

**Supplementary information:**

The online version contains supplementary material available at 10.1007/s00216-023-04852-y.

## Introduction

Liquid chromatography high-resolution mass spectrometry (LC/HRMS) with or without ion mobility (IM) is increasingly used to detect chemicals in environmental waters [1], food [2], and biota [3] as well as biological liquids from humans [4]. Non-target screening (NTS) [5] aims to detect a broad range of chemicals without making a prior selection of target molecules and therefore reduce the possibility of overlooking significant chemicals [6]. The complications in NTS arise in processing the empirical analytical information obtained from the LC/HRMS analysis. The analytical information, such as exact mass of parent ion, isotope pattern, and accurate mass tandem mass spectra (MS^2^ spectra) are used to propose the molecular formula of the detected chemical features [7]. Thereafter, a list of isomeric candidate structures can be obtained by matching the measured MS^2^ spectra with experimental reference spectra or in silico–predicted MS^2^ spectra in libraries [8, 9]. Alternatively, the measured MS^2^ spectra can be converted into probabilistic molecular fingerprints [10], which can be further matched with databases such as PubChem [11]. The long lists of isomeric candidate structures can be reduced by using other analytical information such as LC retention time [12–14] or IM collision cross-section (*CCS*) values [15, 16]. For this purpose, the analytical property is predicted for all candidate structures with a machine learning model and thereafter compared with the experimental property keeping in mind the accuracy of the predictions. The candidate structures that yield predicted retention times or *CCS* values deviating from the experimental ones by more than the confidence interval of the prediction algorithm are considered unlikely. Therefore, the structures are discarded from further consideration or their ranking among the candidate structures is decreased [17]. In addition, metadata, such as usage and previous detection of the candidate structure, can be used to prioritize some candidate structures over others [18]. Ideally, after these steps, the detected feature can be annotated with one most probable candidate structure; however, in practice, multiple isomeric candidate structures remain [19].

Predicting empirical analytical information, such as retention time, from the structure of a candidate chemical is a complicated task due to the complexity of the retention process and data available for modeling. In case of reversed-phase LC, the retention time of the chemicals is dependent on the acid-base chemistry. The retention time depends on the equilibrium between neutral and ionic species [20] and mobile phase pH, which is furthermore dependent on the organic modifier type and percentage in the mobile phase as well as buffers used for the separation [21]. Additionally, retention times tend to vary from one column patch to another due to minuscule changes in the column chemistry [22]. Small factors having a large impact on the retention time make it very hard to accurately model retention time for a diverse set of chemicals. Furthermore, the scarcity and quality of the data available for developing the in silico methods contribute to the prediction accuracy of the models [23]. As authors report different error metrics, it is complicated to directly compare the prediction errors for published models. Still, most retention time prediction algorithms yield a root mean square error (RMSE) around 1.5 min for a 15-min gradient time [13, 14, 24]. The RMSE value is indicative of the prediction accuracy that can be expected from the models when predicting retention times for candidate structures. For *CCS* values, the expected RMSE values exceed 2% [25–27].

To annotate detected features with one correct candidate structure, accurate in silico property prediction algorithms with narrow confidence intervals are required; however, the LC/IM/HRMS also needs to separate the isomeric chemical structures. A standard approach in evaluating the performance of the NTS methods is to spike a sample with a set of known chemicals and evaluate the workflow based on the true-positive rate [28], that is, the fraction of spiked chemicals recovered in the list of candidate structures. The true-positive rate is also used for evaluating in silico property prediction methods [29]. The interpretation of the results, however, is complicated. The spiked chemicals are selected based on the availability of analytical standards and might represent only a fraction of the chemical space of the sample. Therefore, the spiking experiments serve as a proxy for evaluating the true-positive and false-negative rate. Some idea of the false-positive rate may be obtained by looking at studies where the tentative structure candidates are evaluated with analytical standards; e.g., Wang et al. confirmed 133 of 335 tentatively identified chemicals with analytical standards. However, the evaluation of the number of true negatives and false positives for a sample analyzed with LC/HRMS NTS is impossible. Here, false positives denote chemicals that are reported to be present in the sample though they are actually missing while true negatives are chemicals that were not present in the sample and were also not reported.

On the other hand, developing target analytical methods requires thorough method optimization of chromatographic as well as mass spectrometric parameters for high selectivity and sensitivity. Indicating that even if analytical standards are available, the analytical methods and their resolution is of utmost importance in achieving high true-positive and low-false positive rates. This poses two questions. Firstly, to what extent is the false-positive rate in NTS affected by the accuracy and prediction interval of the in silico methods and to what extent by the inability of the NTS LC/IMS/HRMS methods to separate candidate structures? Secondly, how precise would the in silico methods need to be to allow unequivocal structure annotation equivalent to annotation based on analytical standards? Answering these questions in NTS is complicated as the empirical analytical information of the isomeric candidate structures is unknown and analytical standards for the isomeric chemicals are scarcely available.

Here, we assess the efficiency of structural annotation in LC/IMS/HRMS with in silico methods and with analytical standards. As a case study, we focus on five analytical features detected in a surface water sample with NTS LC/HRMS. We analyze the analytical standards for 14 candidate structures with LC/IM/HRMS. In particular, we evaluate the in silico–predicted retention times, database *CCS* values, experimental reversed-phase retention times, experimental *CCS* values from low- and high-resolution ion mobility, and experimental MS^2^ spectra for their ability to yield unequivocal structural annotation. This allows us to (1) separately evaluate the potential for unequivocal structural annotation based on in silico tools and analytical standards as well as (2) assess the prediction accuracy required from the in silico tools to minimally contribute to the false positive rate.

## Materials and methods

### Chemicals

Analytical standards of monuron, diuron, alachlor, acetochlor, 3-(3-chlorophenyl)-1,1-dimethylurea, desethyl-sebuthylazine, desethyl-terbuthylazine, 3-(2,3-dichlorophenyl)-1,1-dimethylurea, 3-(2,4-dichlorophenyl)-1,1-dimethylurea, 3-(2,5-dichlorophenyl)-1,1-dimethylurea, 3-(2,6-dichlorophenyl)-1,1-dimethylurea, and 3-(3,5-dichlorophenyl)-1,1-dimethylurea as well as a standard mixture containing sebuthylazine and terbuthylazine were obtained from Sigma-Aldrich and were of analytical grade or higher.

For the *CCS* calibration nicotinate, adenine, acetaminophen, pyridoxine, caffeine, atrazine, metolachlor, sulfadimethoxine, and ampicillin were used. The calibrants were all purchased from Sigma-Aldrich and were of analytical grade.

Acetonitrile (CHROMASOLV™ for HPLC, gradient grade,  ≥ 99.9%) and water (Honeywell Riedel-de Haën™, Seelze, Germany) with 0.1% of formic acid (reagent grade, 98–100% from Scharlau, Barcelona, Spain) were used as mobile phase for LC/IMS/HRMS analysis.

### LC/HRMS analysis of water samples

To obtain the candidate lists, three different surface water samples, spiked altogether with 152 chemicals (mostly pesticides, pharmaceuticals, and other known environmental contaminants), were analyzed with LC/ESI/HRMS, as described previously by Peets et al. [30]. In short, measurements were carried out on Thermo Scientific Dionex Ultimate 3000 (Thermo Fisher Scientific, USA) and Thermo Scientific Q Exactive Orbitrap. The samples were separated with a Kinetex 2.6-µm EVO C18 150 × 3.0 mm reversed-phase column (Phenomenex, Torrence, CA, USA). The mobile phase consisted of 0.1% formic acid and acetonitrile. The acetonitrile content in the mobile phase was increased from 5 to 100% over 20 min, kept at 100% for 5 min, and finally returned to 5% over 0.1 min. Equilibration time between injections was 5 min. The column oven temperature was 40 °C, and the mobile phase flow rate was 0.350 mL/min. All measurements were carried out in positive ESI mode, and the spray voltage was set to 3.5 kV while max spray current was 100 μA. Auxiliary gas, sheath gas, and sweep gas flow rates were 3, 35, and 0 arbitrary units, respectively, while the auxiliary gas and capillary temperature were both 320 °C. S-lens RF level was 50%. The resolution was set to 120,000, AGC target was 3,000,000, maximum IT was 200 ms, and two full scans (60.0000–900.0000 m/z and 100.0000–1500.0000 m/z) were acquired in centroid mode. The MS^2^ spectra were acquired in data-dependent mode with an inclusion list. The number of microscans was 1; resolution, 30,000; AGC target, 1 × 10^5^; and maximum IT, 60 ms. Stepwise normalized collision energies of 20, 70, and 120 were used for fragmentation.

### Obtaining candidate structures with SIRIUS-CSI:FIngerID

Structure candidate lists were generated with SIRIUS + CSI:FingerID version 4.9.5 [10]. For compounds with a molecular weight below 1000 Da, the highest number of atoms was set as follows: carbon, hydrogen, oxygen, nitrogen, and fluorine were set to infinity, chlorine: sixteen, sulfur: twelve, boron: eleven, bromide: ten, silicon: nine, phosphorus: eight, iodine: six, arsenic: two, and selenium: two. For those with a molecular weight between 1000 and 2000 Da, it was as follows: chlorine: eighteen, bromine: fifteen, silicon: ten, and selenium: four, and the remaining elements were set as for molecules below 1000 Da. Based on the instrumental mass accuracy, the mass error tolerance was set to 5 ppm. Candidate structures were obtained by matching the molecular fingerprints predicted by SIRIUS + CIS:FingerID with the fingerprints of chemicals in all databases available in SIRIUS.

### In silico fragmentation for candidate structures

The 25 top candidate structures per feature from SIRIUS+CSI:FingerID were considered, and for all candidate structures, the in silico MS^2^ spectra were computed with CFM-ID [31] at low (10 V), medium (20 V), and high (40 V) collision energy. The three spectra were combined, and the cosine similarity with the experimental MS^2^ spectra was computed with an in-house script in R. For all spectra, the number of fragments in the in silico spectra was significantly higher than the number of fragments in the experimental spectra, yielding low cosine similarity for the majority of the candidate structures. Generally, a few peaks in the experimental spectra were explained by the in silico–predicted MS^2^ spectra; at least one common peak was required for considering the isomeric candidate structure. The final selection of the candidate structures for experimental testing was limited by the commercial availability and in total 14 chemicals (see “Chemicals” and Table 1) corresponding to five LC/HRMS features.

**Table 1:** Overview of the experimental analytical characteristics of the studied isomers, retention times predicted with MultiConditionRT [24], database CCS values from PubChem [11], and log*P* values predicted with ChemAxon [34]. For *CCS* values, multiple values are given if the chemical has been characterized by several research groups and PubChem includes multiple values. For predicted retention times, the confidence interval is given based on the two times RMSE of the model validation by Souihi et al. [24]

Molecular formula	Name	RT (min)	RT_predicted_ (min)	*CCS* (Å^2^)	*CCS*_database_ (Å^2^)	log*P*
C_9_H_11_ClN_2_O	Monuron	7.0*	8.0 ± 3.1	142.0*		1.9
	3-(3-Chlorophenyl)-1,1-dimethylurea	7.2*	8.2 ± 3.1	142.4*		2.0
C_14_H_20_ClNO_2_	Alachlor	11.4*	11.5 ± 3.1	158.6*	156.41	3.5
	Acetochlor	11.5*	11.4 ± 3.1	159.3*	157.40, 160.09, 173.50	3.0
C_9_H_16_ClN_5_	Sebuthylazine	9.9	12.7 ± 3.1	153.7*		2.6
	Terbuthylazine	9.4	13.7 ± 3.1	153.2*		3.2
C_7_H_12_ClN_5_	Desethyl-sebuthylazine	6.3	8.1 ± 3.1	144.3*		1.7
	Desethyl-terbuthylazine	7.2	7.5 ± 3.1	144.3*	144.71	1.8
C_9_H_10_Cl_2_N_2_O	Diuron	8.6*	9.4 ± 3.1	149.1*	148.38, 150.00, 152.30, 153.32	2.5
	3-(2,3-Dichlorophenyl)-1,1-dimethylurea	8.4*	9.6 ± 3	145.5*		2.5
	3-(2,4-Dichlorophenyl)-1,1-dimethylurea	8.6*	9.2 ± 3.1	146.3*		2.5
	3-(2,5-Dichlorophenyl)-1,1-dimethylurea	8.9	9.6 ± 3.1	146.4*		2.5
	3-(2,6-Dichlorophenyl)-1,1-dimethylurea	5.8	9.3 ± 3.1	143.6*		2.5
	3-(3,5-Dichlorophenyl)-1,1-dimethylurea	9.2	9.1 ± 3.1	149.9*		2.5

*Isomeric candidate structures that could not be resolved with reversed-phase chromatography or low-resolution IM

### LC/IMS/HRMS analysis of candidate structures

The surface water samples were analyzed with an LC/Orbitrap; however, to evaluate the feasibility of structural assignment based on ion mobility, further investigation of the commercially available candidate structures was achieved with Waters Select Series Cyclic IMS. Chromatography was performed on an analytical column, Kinetex PS C18 100 Å, 150 mm × 3 mm id, 2.6 µm particle size, purchased from Phenomenex. The column temperature was set to 30 °C, and the injection volume was 10 µl. The mobile phase was a combination of water with 0.1% formic acid (A) and acetonitrile (B). For LC separation on column, a gradient elution program at 0.35 mL/min flow was used as follows: 15% acetonitrile ramped to 95% linearly over 15 min then held for an additional 5 min. After 20 min, with the same flow, the mobile phase was returned to 15% acetonitrile over the course of 0.1 min and equilibrated for 3 min. For ion mobility measurements, both chromatography as described and flow injection analysis at 0.2 mL/min with a mobile phase containing 20/80 0.1% formic acid and acetonitrile were utilized.

Ion mobility and high-resolution mass spectrometry measurements were carried out on a Waters Select Series Cyclic IMS platform with time-of-flight mass analyzer operated in V-mode. In electrospray ionization (ESI), the capillary voltage, cone voltage, and source offset were adjusted to 1.80 kV, 40 V, and 10 V, respectively. For LC/HRMS experiments, the source temperature and desolvation temperature were set to 150 °C and 550 °C. For infusion experiments, the source temperature was 100 °C, desolvation temperature was 400 °C, and desolvation gas flow was 600 L/h. Nebulizer gas and reference capillary voltage were held at 6 bar and 1.50 kV, respectively. Leucine enkephalin with a reference *m/z* of 556.27658 in positive ESI was infused into the source during both the LC analysis and flow injections and used to lockmass-correct all measured *m/z*.

In full-scan experiments and ion mobility experiments, the trap CE and transfer CE were kept at 6 V and 4 V and post-trap gradient and post-trap bias were 7 V and 35 V for all measurements. The drift cell Twave velocity was 375 m/s, and the Twave height was set to 10 V for all ion mobility measurements except multiple cycle experiments where 12 V was used.

The time-of-flight mass analyzer operated in V-mode with resolution of 76,600 and mass accuracy of 5 ppm or better. The full-scan spectra were recorded from *m/z* of 50 to 300 Da. The low-resolution IM separation used for the determination of *CCS* values was based on a single pass cyclic sequence (i.e., one cycle). The sequence was as follows: 10 ms “inject,” 2 ms “separate,” and 39.6 ms “eject and acquire” (allowing for 3 pushes per bin). All voltages used within these cyclic functions were kept at default values except for the “array TW height” which was lowered to 10 V during “eject and acquire” in order to prevent fragmentation. For multiple pass experiments, i.e., separation of ions with multiple cycles in IMS, the same cyclic sequence was used, but here, the separation time was adjusted to achieve the desired number of cycles for each analyte group. The resolution obtained depends on the number of passes as well as on the analyte. For example, resolution of  ~ 15 was observed for three-pass experiments, where resolution is calculated as d*t*/Δd*t*, where d*t* is the drift time of the ion mobility peak and Δd*t* is the drift time difference of the two ion mobility peaks.

The visualization of the extracted ion chromatograms, driftograms, and MS^1^ and MS^2^ spectra was done in R (version 4.2.1) based on raw data extracted with MassLynx in a.csv format.

### *CCS* calibration

Nine chemicals (see “Chemicals”) with experimentally known *CCS* values from Picache et al. [32] were selected for *CCS* calibration and analyzed with the IM settings described in “LC/IMS/HRMS analysis of candidate structures.” These chemicals represent common small contaminants and metabolites with *CCS* values from 123.9 to 192.4 Å^2^; therefore, these standards are more similar to the isomeric structural candidates studied here than the commonly used polyalanine standards. Still, these standard chemicals are not homologous series or isomers of the analytes as no such analytical standards with known *CCS* values are commercially available. To account for the heterogeneity of the structure of the calibration standards, we include the uncertainty arising from the calibration graph in the uncertainty budget of the *CCS* values (“Uncertainty of CCS values”).

For obtaining the *CCS* values, the ion mobility trace corresponding to the MS trace of the exact mass of the parent ion was extracted in MassLynx 4.2 (Waters Corporation, UK) and the Gaussian peak fitting was performed in R with an in-house script. The process was repeated for all isomeric candidate structures as well as *CCS* calibrants. Linear regression was thereafter used to compute the *CCS* values for the isomeric candidates.

### Uncertainty of *CCS* values

The uncertainty in the experimentally determined *CCS* values was estimated based on the Nordtest approach [33] and accounts for both the systematic component as well as a random component. The systematic component is primarily the imperfect fitting of a straight line through *CCS *vs drift time plot and was calculated as the square root mean of relative residuals of *CCS* values of the calibration chemicals from the regression analysis of the *CCS* vs drift time. The random component arises from the daily variation in the measured *CCS* values and was estimated as the relative pooled standard deviation of predicted $\widehat{CCS}$ values for the calibration chemicals from measurements on 4 days over a period of 1 month. The systematic and random component were combined:

1 $$\frac{u\left(CCS\right)}{CCS}=\sqrt{\frac{1}{n}\sum\nolimits_{i}^{n}\frac{{\left({\widehat{CCS}}_{i}-{CCS}_{i}^{\mathrm{ref}}\right)}^{2}}{{CCS}_{i}^{\mathrm{ref}}}+\frac{1}{n}\frac{1}{m}\sum\nolimits_{i=1}^{n}\sum\nolimits_{j=1}^{m}\frac{{\left({CCS}_{i,j}-{\overline{CCS} }_{i}\right)}^{2}}{{\overline{CCS} }_{i}}}$$

where *i* is the number of calibration chemicals and *j* is the number of days. In combination, the measurement uncertainty of *CCS* values 0.54% (*k* = 2) was observed. Furthermore, the relative standard deviation of the *CCS*^ref^ of the calibrants ranged from 0.01 to 1.14%, leading to an expanded uncertainty *U*(*CCS*^ref^) of 0.02 to 2.28% (*k* = 2). Here, we take the worst case scenario and assume that the expanded uncertainty *U*(*CCS*^ref^) is 2.28%, yielding a combined uncertainty of 2.3% (*k* = 2).

Additionally, when matching experimental and database *CCS* values, the uncertainty in the database *CCS* values needs to be accounted for. Unfortunately, for the majority of the database *CCS* values, respective uncertainties are not reported. Therefore, we assume here that the uncertainty of the database entries is comparable with the experimentally determined uncertainty here (2.3%, expanded uncertainty). Combining the uncertainty of both experimentally determined CCS values and database values yields a combined uncertainty (*k* = 2):

2 $$\sqrt{{U\left({CCS}_{\mathrm{exp}}\right)}^{2}+{U\left({CCS}_{\mathrm{database}}\right)}^{2}}=\sqrt{{2.3\%}^{2}+{2.3\%}^{2}}=3.25\%$$

### Predicting retention time and obtaining reference *CCS* values

For in silico structure annotation, the retention times were predicted with MultiConditionRT by Souihi et al. [24] and the *CCS* values were retrieved from PubChem database [11]. The log*P* values were estimated with ChemAxon [34] and molecular descriptors with PaDEL descriptor calculator [35]. The uncertainty in the predicted retention times was estimated from the original publication by Souihi et al. [24]. Moreover, the uncertainty in the predicted log*P* values as well as molecular descriptors contributes to the accuracy in the predicted retention time. In short, the retention times were predicted for fourteen chemicals and the root mean square error (RMSE) of these predictions, 1.55 min, is used as an estimate of the standard uncertainty (*k* = 1) of the predicted retention times. The expanded uncertainty was therefore 3.1 min (*k* = 2).

Agreement between the experimental *CCS* values from this study and database values was evaluated on the basis of normalized error *E*_n_ value:

3 $${E}_{\mathrm{n}}=\frac{{CCS}_{\mathrm{reference}}-{CCS}_{\mathrm{measured}}}{\sqrt{{U}_{\mathrm{reference}}^{2}+{U}_{\mathrm{measured}}^{2}}}$$

*E*_n_ considers the uncertainty from both reference and measured CCS value. An *E*_n_ below 1 means that the experimental value and reference value agree within the uncertainty limits. An *E*_n_ above 1 indicates that they do not agree.

## Results and discussion

**Fig. 1:**
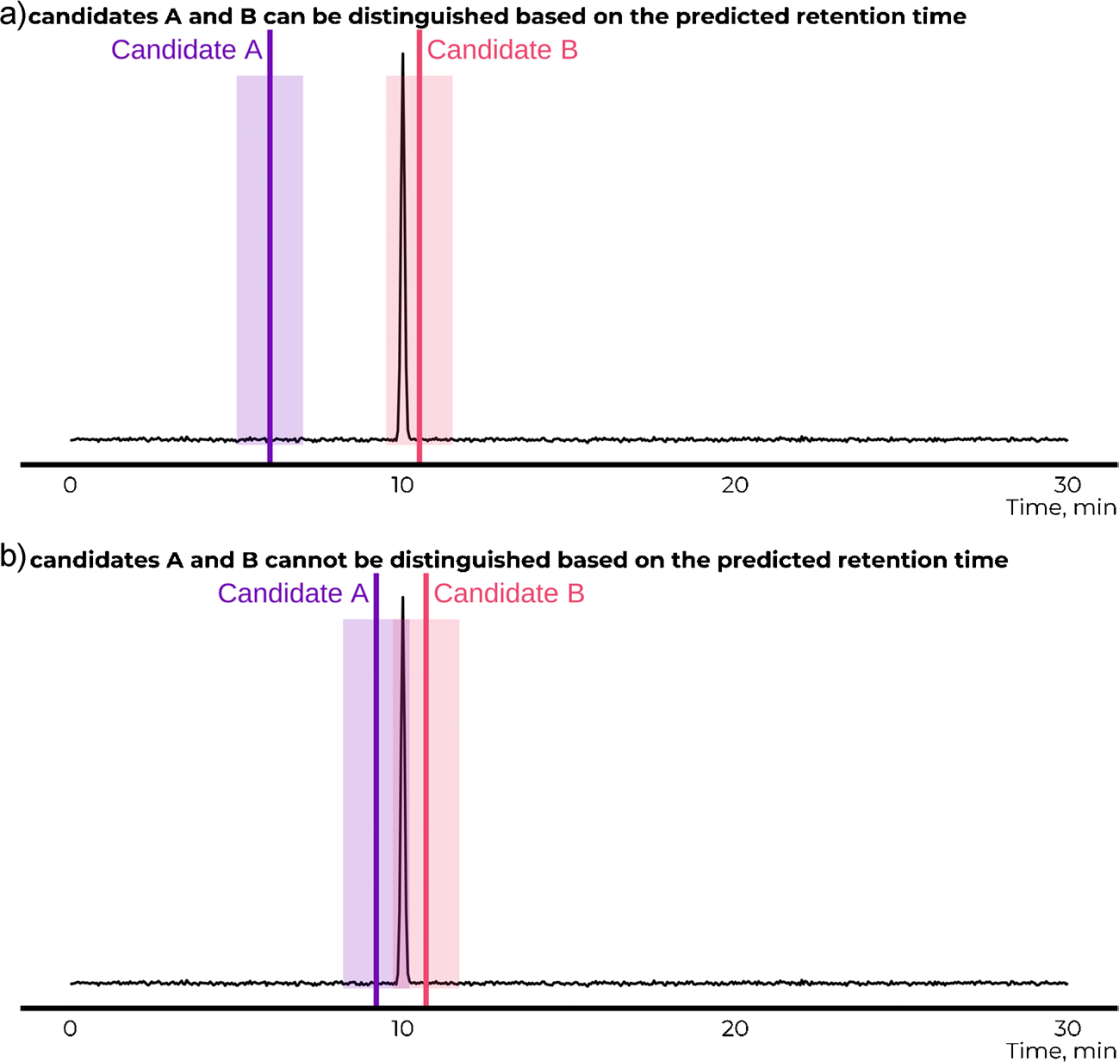
The predicted retention times of candidate structures A and B are shown as horizontal lines while the prediction interval is indicated as a rectangle. **a** The experimental retention time overlaps with the predicted retention time for the candidate structure B but not for candidate structure A. The candidate structure A can be deemed less likely. **b** The predicted retention times for both candidate structures A and B overlap with the experimental retention time, and neither of the candidate structures can be deemed unlikely

The ambiguity in structural identification can arise from (1) limitations of the resolution of the instrumental methods or (2) accuracy of the in silico models used for confirming, ruling out, or re-ranking the candidate structures. For example, given an LC peak with retention time 10.0 min and two candidate structures with predicted retention times of 9.2 ± 1.0 min and 10.7 ± 1.0 min, both candidate structures agree with the experimental retention time within the prediction interval. Therefore, it becomes impossible to rule out one or both of the candidate structures due to the width of the confidence interval of the in silico–predicted retention times (Fig. 1). This limitation persists until the confirmation with the analytical standards is available even if the two candidate structures would actually be separated in LC.

On the other hand, isomers that yield identical retention time in LC column and marginally different fragmentation spectra in HRMS cannot be distinguished even if analytical standards are available. In this case, the limitations arise on the instrumental/method level. Therefore, the accuracy of structural annotation is related to the accuracy of the machine learning model used for in silico prediction of the empirical analytical information for candidate structures but also on the ability of LC/HRMS to separate the candidate structures.

Improving the in silico prediction models for retention time [13, 14, 17, 36] and collision cross sections [16, 26] has received increasing attention in the NTS community, while the limitations of the instrumental methods have been discussed to a lesser extent. Still, acknowledging the ambiguity of the structural assignment resulting from the instrument/method resolution is essential to understanding the limitations of NTS. Additionally, pinpointing analytical information that provides the highest separation of isomeric structures can help to focus the attention of developing in silico methods. The analytical dimension that is able to separate more isomeric candidate structures can also enable ruling out more of the candidate structures, given sufficient performance of the in silico methods.

### In silico structure annotation

The retention times were predicted for all fourteen candidate structures with the MultiConditionRT [24] algorithm recently developed in our group. MultiConditionRT accounts for the structure of the candidate as well as the mobile phase composition. In most cases the uncertainty of the predicted retention times of the candidate structures was greater than the margin in the experimental retention times of the isomeric candidate structures. Even for the most diverse group where retention times were observed to vary over more than 2 min, the predicted retention times differed by less than 0.5 min and did not necessarily follow the experimental retention order (Table 1). For four of the features, the in silico–predicted retention times did not allow ruling out any of the candidate structures. An exception here was the feature C_9_H_16_ClN_5_ where two candidate structures, sebuthylazine and terbuthylazine, were investigated. The experimental retention times for the two structures were 9.9 and 9.4 min. Terbuthylazine is the correct candidate structure for the LC/IMS/HRMS peak with a retention time of 9.4 min; however, the in silico–predicted retention time is 13.7 ± 3.1 min. Therefore, the interval of the predicted retention time does not overlap with the retention times of the investigated LC/IMS/HRMS feature. This mismatch would result in deeming terbuthylazine as an unlikely candidate structure, hence a false-negative assignment. Similarly, the experimental retention time of 3-(2,6-dichlorophenyl)-1,1-dimethylurea feature differed considerably both from the retention time of the other candidate structures and from that of the correct candidate structure, therefore contributing to another false-negative identification.

The false-negative identifications are likely arising from the high importance of the log*P* in the employed retention time prediction algorithm [24]. For four features, the predicted retention order of the isomeric candidate structures was contradictory to the experimental retention order as well as to the order in predicted log*P* values. Furthermore, all candidate structures of C_9_H_10_Cl_2_N_2_O have the same log*P* value and also the predicted retention times are very similar. On the other hand, the experimental retention times vary significantly with the position of the chlorine group. This indicates that the retention time prediction model has learned the general association between reversed-phase liquid chromatography retention time and log*P* values but is unable to account for the subtle structural differences in the isomeric structures, which complicates the structural assignment of candidate structures, which are isomeric to each other.

In order to evaluate the possibility of structural annotation based on *CCS* values, the measured *CCS* values were compared with the available *CCS* values in the PubChem database. The experimentally determined *CCS* values as well as values in the databases are subject to uncertainty arising from fitting a Gaussian peak to the ion mobility trace, fitting a linear regression to calibration graph (*CCS* vs drift time), as well as repeatability of the measurements. Combining these uncertainty sources, an expanded relative uncertainty *U*(*CCS*) of 2.3% was observed for the experimental *CCS*_exp_ values. This means that with 95% probability, the reference *CCS* value should be within the confidence interval *CCS*_exp_ ± *U*(*CCS*). For comparison, the uncertainty in the database CCS values needs to be accounted for, which leads to the combined uncertainty of 3.25%. Therefore, candidate structures with database *CCS* values more than 3.25% different from the experimental *CCS* values of the detected feature can be deemed unlikely.

Reference *CCS* values were available for four candidate structures: alachlor, acetochlor, diuron, and desethyl-terbuthylazine. Firstly, the database values from different sources did not agree with each other perfectly. Acetochlor has three different *CCS* values (173.50, 160.09, and 157.40 Å^2^) in PubChem, and the difference between the reference values was significant. In the first set of values, a lower *CCS* value was reported for the sodium adduct of acetochlor (159.1 Å^2^), indicating a clear inconsistency with the other sets, and was considered here as a possible false assignment. Secondly, for three of the structure candidates, the experimental *CCS* values agreed with the database values within the confidence interval. Unfortunately, the database *CCS* values also agreed with the *CCS*_exp_ values of two or more isomeric candidate structures (Table 1). Furthermore, the agreement was equally good for database *CCS* values measured on traveling wave and drift tube instruments. This indicates that the unequivocal structure annotation based on database *CCS* values would be impossible. Only 3-(2,6-dichlorophenyl)-1,1-dimethylurea could be distinguished from diuron and 3-(3,5-dichlorophenyl)-1,1-dimethylurea based on a significantly lower *CCS* value than the experimental CCS values for diuron (difference of 3.8% and 4.4%). This clearly highlights the need to reduce the uncertainty in the database values as well as in experimental CCS values.

The database *CCS* values were only available for four of the 14 candidate structures. Therefore, one could also predict the *CCS* values with different machine learning algorithms [25, 26]. However, for predicted CCS values, the confidence interval is even larger than for the database values and therefore they were not separately evaluated.

### Structure annotation with analytical standards

**Fig. 2:**
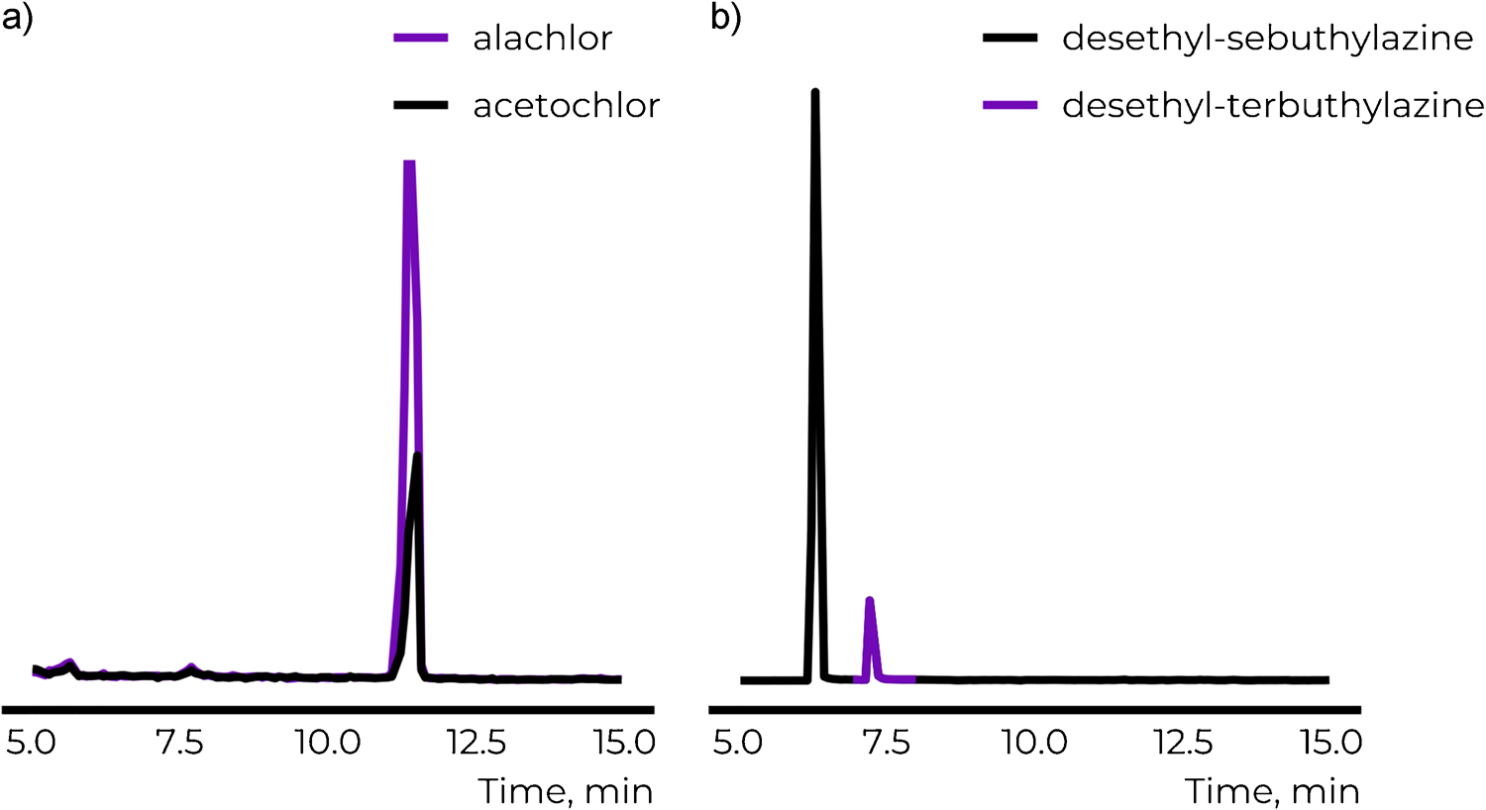
Out of the studied isomeric candidate structures, **a** alachlor and acetochlor showed the poorest chromatographic separation with completely overlapping peaks and **b** desethyl-sebutylazine and desethyl-terbutylazine showed the best separation

To evaluate if unequivocal structural annotation is possible if analytical standards are available, all fourteen candidate structures were analyzed with LC/IM/HRMS. The isomeric candidate structures showed similar retention times; mostly a difference of less than a minute was observed (Table 1, Figs. S1-S5). The peaks of alachlor and acetochlor, two isomeric candidate structures that differ in the position of one methyl group, completely overlapped (Fig. 2) while peaks of monuron and 3-(3-chlorophenyl)-1,1-dimethylurea partially overlapped. For the isomeric structures of C_9_H_10_Cl_2_N_2_O, the peaks of three candidate structures partially overlapped. Two more isomeric candidate structures eluted within 1 min from the group but were baseline separated. Nevertheless, 3-(2,6-dichlorophenyl)-1,1-dimethylurea showed more than 2 min shorter retention time than the other candidate structures (Fig. S5). The peaks of terbutylazine and sebutylazine were baseline separated (∆*t*_R_ of 0.4 min, Fig. S2) as were desethyl-sebutylazine and desethyl-terbutylazine (∆*t*_R_ of 0.9 min, Figs. 2 and S3). Therefore, for four out of five LC/HRMS features, unequivocal structure annotation is possible based on retention times if analytical standards are available for comparison.

To evaluate the structural assignment of the isomeric candidate structures based on IM, a mixture of isomeric candidate structures was analyzed with direct infusion with cyclic ion mobility. In the low-resolution mode, a single ion mobility cycle was used for the separation. For unequivocal annotation based on the IM, at least partial resolution of the candidate structures is required. Unfortunately, isomeric candidate structures appeared as one unresolved mobility peak. This indicates that the studied isomers have close drift times and a short drift path is unable to separate these structures.

**Fig. 3:**
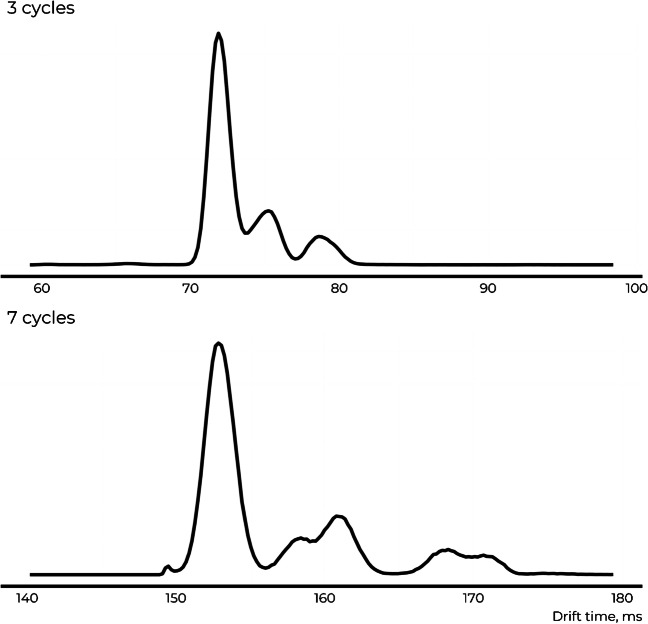
Increasing the number of cycles used for ion mobility separation increases the resolution of the candidate structures for six candidate structures of C_9_H_10_Cl_2_N_2_O; however, none of the isomeric candidate structures studied here could be baseline separated from the others

The separation was improved by increasing the number of passes, here referred to as high-resolution ion mobility separation. The number of passes was optimized based on the group of isomers and ranged between 7 and 33 cycles; however, none of the groups of isomeric candidate structures could be baseline separated (Figs. 3 and S6-S10). For example, the separation of monuron and its isomer increased by increasing the number of passes up to 29 without reaching the baseline separation. Increasing the number of passes further did not improve the separation (Fig. S9).

By increasing the number of passes, the separation time also increases and the sensitivity decreases. For partial separation of alachlor and acetochlor, 33 separation cycles and  ~ 750 ms of separation time was required. Simultaneously, the width of the chromatographic peaks was around 10 s. This indicates that increasing the number of cycles reduces the points per peak and increases the likelihood of overlooking important chromatographic features. Therefore, multi-pass IM methods can be deemed better suited for further investigation of interesting features after the preliminary analysis.

Fragmentation spectra are an essential starting point for obtaining the molecular formula and candidate structures in NTS LC/HRMS. It was of interest to evaluate if an unequivocal candidate structure can be suggested based on the MS^2^ spectra. Here, four out of five groups of isomers yielded fragments with the same *m/z*. One of the groups of isomeric candidate structures, acetochlor and alachlor, could be distinguished based on the MS^2^ spectra as main fragments showed a mass difference corresponding to a –CH_3_ group (Fig. S11). Terbutylazine and sebutylazine as well as desethyl-sebutylazine and desethyl-terbutylazine yielded fragments with the same exact mass but with significantly different peak ratios (Fig. S12). For monuron and 3-(3-chlorophenyl)-1,1-dimethylurea as well as for diuron and its five isomers, all observed fragments overlapped and the isomeric candidate structures remained indistinguishable based on the peak ratios (Figs. S13 and S14). For some groups, significant differences were observed only at low (15 V) or high (40 V) collision voltages but not at both. The intensity ratio is important in identifying the chemicals in comparison with spectra obtained with analytical standards and has been suggested as an identification criterion for targeted analysis in validation guides such as SANTE [28]. However, intensity ratios are more complex to incorporate in the structural identification in NTS when comparing experimental spectra with database spectra. The internal energy given to the molecule during fragmentation cannot be directly compared between different instruments due to differences in the instrument geometry and even units (absolute vs relative) used for reporting fragmentation energy. As a result, comparing reference spectra with the experimental spectra is straightforward if the reference spectrum has been registered on the same instrument. This applies, for example, if analytical standards are available or experimental spectra are compared with vendor databases. This complication, however, does not reduce the importance of the community efforts in assembling databases of MS^2^ spectra. Such collections have high value in comparing the *m/z* value of the fragments as well as providing a data source for training accurate in silico models for predicting spectra or predicting molecular fingerprints from spectra.

All in all, retention time proved to be the most powerful analytical characteristic for structural annotation of isomeric candidate structures given the availability of the analytical standards. Noteworthily, the unequivocal structural assignment was possible even if the log*P* values of the candidate structures were indistinguishable, as in the case of the candidate structures for C_9_H_10_Cl_2_N_2_O, where three out of six candidate structures could be baseline resolved, though the predicted log*P* values were identical. This indicates that retention time is a very powerful empirical analytical characteristic in structural assignment. The only set of isomeric candidate structures that could not be distinguished based on retention time was clearly distinguishable based on the MS^2^ spectra.

### Perspective

Though retention time was a very powerful tool for structural assignment given the availability of analytical standards, in all but one case the chromatographic peaks eluted within 1 min. Depending on the algorithm used for predicting the retention time, the confidence intervals vary from one to a few minutes for a 15-min gradient profile [24]. As a result, in spite of chromatographic separation, distinguishing between the isomeric candidate structures based on the predicted retention times was ambiguous.

From the group of isomeric chemicals investigated here, the most critical are the group of monuron and diuron as *CCS* values and MS^2^ spectra provide very little identification power. The retention time difference in these chemicals is around 0.2 min. Highlighting that the accuracy of the retention time prediction algorithms would need to improve significantly to reduce the false-positive rate and complement the high separation power achievable in LC.

**Fig. 4:**
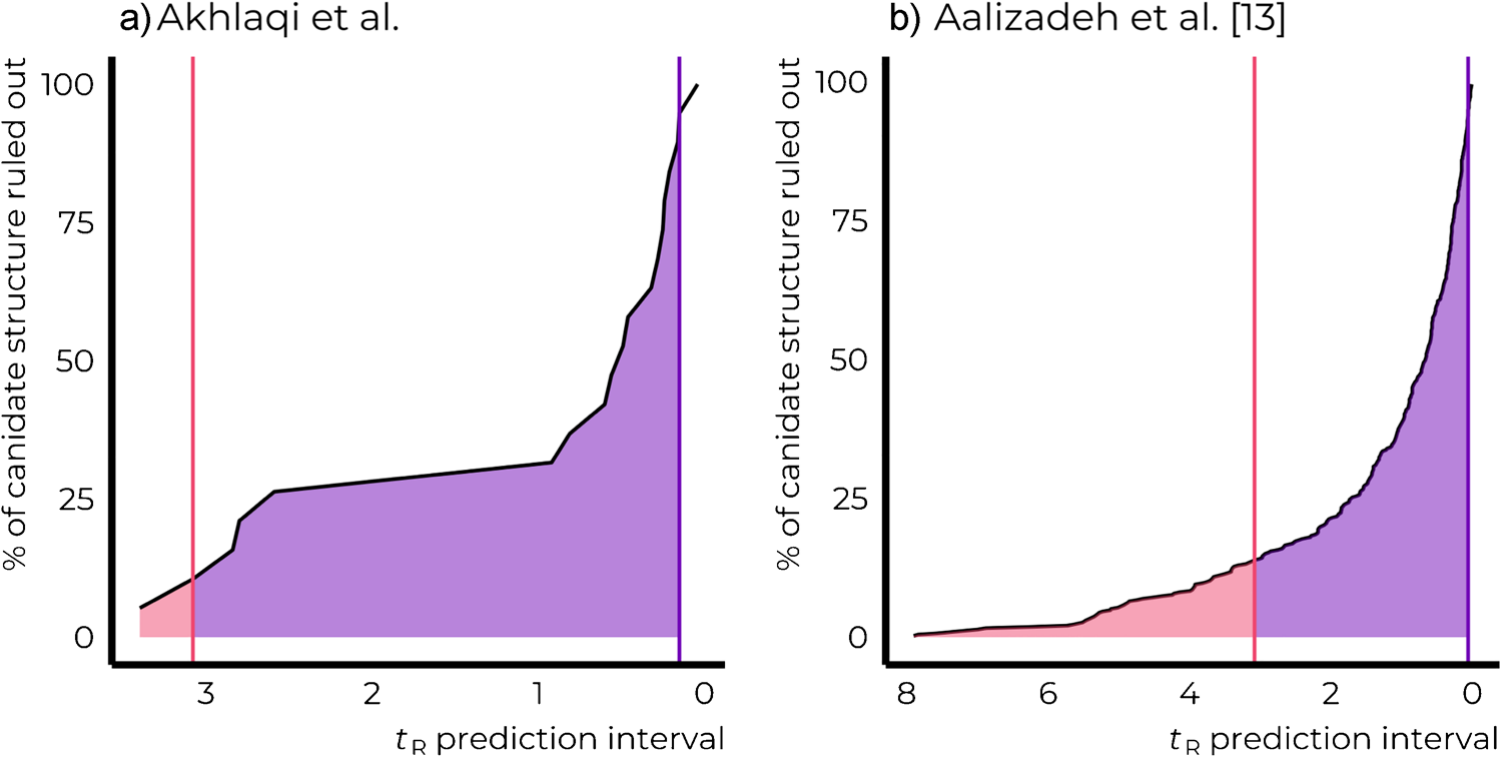
Only **a** 10.5% (current study) to **b** 13.6% (Aalizadeh et al. [13]) of the pairs of isomeric candidate structures could be distinguished based on the uncertainty margin of the current retention time prediction models (pink line)

To give an indication of prediction accuracy required from the in silico method to distinguish between isomeric candidate structures, we evaluated the absolute retention time differences for each pair of isomers studied here. The median retention time difference of 0.5 min was observed, indicating that for distinguishing half of the isomer pairs, the width of the retention time prediction interval would need to be below 0.5 min (± 0.25 min). To distinguish 95% of the isomeric chemicals in this dataset, a retention time prediction interval narrower than 0.15 min (± 0.075 min) would be required. Due to the limited number of chemicals studied here, we furthermore evaluated the retention time differences for isomeric chemicals studied by Alygizakis et al. [13]. Similarly to the current study, only 13.6% of the 432 pairs of isomeric structures were in silico distinguishable given the expanded uncertainty of 3.1 min of the retention time prediction model [24]. In the case of this dataset, a retention time prediction interval narrower than 0.05 min (± 0.025 min) would be required to distinguish between 95% of the pairs of isomers (Fig. 4).

The challenge in improving the retention time predictions is associated with the difficulty in modeling the retention of the analyte unexplainable by log*P*. Expectedly, the retention times of the chemicals studied here correlated with log*P* (*R*^2^ of 0.66). The separation was carried out in reversed-phase chromatography, and the retention process is associated with the hydrophobicity of the chemicals. Still, the retention order within the groups of isomeric candidate structures did not necessarily follow the log*P* order, indicating the importance of other processes in addition to polarity-based partitioning. For example, acid-base properties of the analyte determine the equilibrium between neutral and cationic/anionic species; which in turn affect the interaction of the analyte with the stationary and mobile phase [20]. Furthermore, two mobile phases with the same pH, same organic modifier and gradient profile but different buffer type are known to yield both different retention times and retention order [20] while stationary phase chemistry varies from manufacturer to manufacturer and even between batches [22]. The large number of variables of a chromatographic method leads to a need for a large number of high-quality experimental data for successful modeling of retention time; therefore, it is unlikely that current strategies would lead to a universal retention time or retention order prediction model any time soon. Furthermore, this highlights the need and potential for in-sample predictions. Recently, Bach et al. [17] proposed a graph-based retention order modeling that allows for re-ranking the candidate structures if structures ranked lower based on MS^2^ spectra match better with the retention time order. Still, the training of these models relies on currently available datasets in reversed-phase liquid chromatography and its ability to pick up properties affecting the retention.

**Fig. 5:**
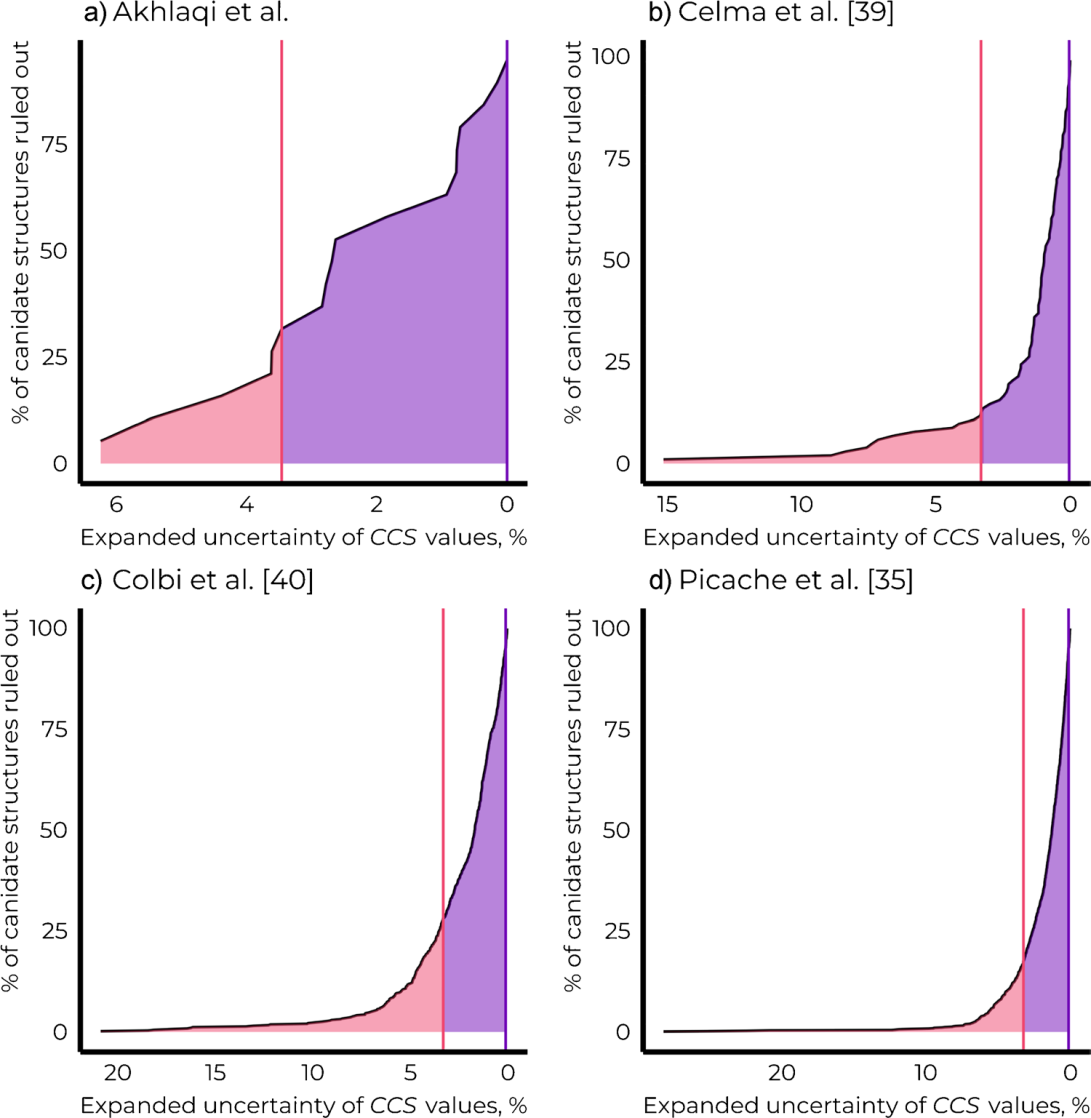
The current expanded uncertainty of *CCS* values enables distinguishing between 12.6 and 31.6% of the pairs of isomeric chemicals, depending on the dataset. The expanded uncertainty of the experimental *CCS* values evaluated here is shown with a pink line, the proportion of pairs of isomeric chemicals that can be distinguished based on the current expanded uncertainty is shown in pink, and the remaining not yet distinguishable proportion is shown in violet

To further evaluate the uncertainty/prediction interval needed to distinguish between the isomeric candidate structures based on *CCS* values, we compared the experimental *CCS* values of isomeric chemicals studied here as well as isomeric chemicals studied previously by Celma et al. [37], Colby et al. [38], and Picache et al. [32]. We observed that 31.6%, 12.6%, 28.0%, and 17.5% of the pairs of isomeric chemicals could be distinguished based on *CCS* values while accounting for the uncertainty margin of 3.25% estimated in this study (Fig. 5). Furthermore, to distinguish between 95% of the pairs of isomeric chemicals, expanded uncertainty as low as 0.04 to 0.15% was required, depending on the dataset. This highlights that extremely accurate *CCS* prediction algorithms are required to distinguish between the isomeric candidate structures.

In spite of the challenging prediction accuracy requirements for each of the individual prediction algorithms, the NTS does not need to rely on only one type of analytical information. As shown above, the pair of isomers that could not be distinguished based on retention time yielded different MS^2^ spectra and could still be structurally annotated. Therefore, a promising avenue in structural annotation seems to be in combining the predictions from multiple separation dimensions and accompanying in silico property prediction models.

## Conclusions

Here, we have evaluated the ability of in silico prediction models and LC/IM/HRMS instrumental methods to distinguish the isomeric candidate structures in NTS. We observed that liquid chromatography retention times provided the highest possibility for structural identification, given the availability of the analytical standards. This was achieved due to the fact that four out of five groups of isomeric candidate structures were chromatographically separated in a reversed-phase liquid chromatography. Still, the current prediction accuracy in the machine learning models for predicting retention time yielded overlapping prediction intervals. Therefore, in silico, most of the features could not be unequivocally annotated and the full advantage of the chromatographic separation remains inaccessible. In contrast, low-resolution IMS provided no separation of the tested groups of isomers. We observe that MS^2^ spectra, which are indispensable for creating the tentative candidate lists, add additional confirmation to one feature that was not separated in liquid chromatography. Hence, combining different analytical characteristics yielded an enhanced possibility for structural annotation of all investigated features, given the availability of the analytical standards.

Additionally, we for the first time evaluated the prediction intervals needed for successful in silico retention time and *CCS* prediction algorithms. Based on the chemicals studied here and previously in literature, we observed that the prediction interval of retention time needs to be 0.05 min or better and of *CCS* values 0.15% or better to distinguish between 95% or the pairs of isomers studied here. By this, we set a clear numerical goal for the in silico prediction algorithm. As this is unlikely to be achievable for any one of the prediction algorithms, the combination of prediction of different analytical characteristics might be desirable.

## Supplementary Information

Below is the link to the electronic supplementary material.

Supplementary file1 (PDF 890 KB)
