# Supplementary material for: Complementary methods for structural assignment of isomeric candidate structures in non-target liquid chromatography ion mobility high-resolution mass spectrometric analysis
Source: Anal Bioanal Chem. 2023 Jul 15;415(21):5247–59. doi: 10.1007/s00216-023-04852-y (PMC10404200; doi:10.1007/s00216-023-04852-y)
Supplement: Supplementary file 1 — Supplementary file1 (PDF 890 KB) [file 216_2023_4852_MOESM1_ESM.pdf]

Supporting Information

Complementary methods for structural assignment of isomeric candidate structures in non-target liquid chromatography ion mobility high resolution mass spectrometric analysis

Masoumeh Akhlaqi,[a] Wei-Chieh Wang,[a] Claudia Möckel,[a] Anneli Kruve[a*]

[a] Department of Materials and Environmental Chemistry, Svante Arrhenius väg 16C, 114 18 Stockholm, Sweden

Chromatographic separation of isomeric chemicals

The chromatographic separation of the candidate structures was studied on a Kinetex PS C18 100 Å column, 150mm×3 mm id, 2.6 µm particle size (Phenomenex) with gradient elution 15% acetonitrile ramped to 95% linearly over 15 min then held for an additional 5 min at a flow rate of 0.35 mL/min. The aqueous phase consisted of 0.1% formic acid. For detection a Waters Select Series Cyclic IMS with Time of Flight mass analyzer in full scan mode was used. Analytical standards of the isomeric candidate structures were injected individually as well as in a mixture to evaluate the retention time and chromatographic resolution.

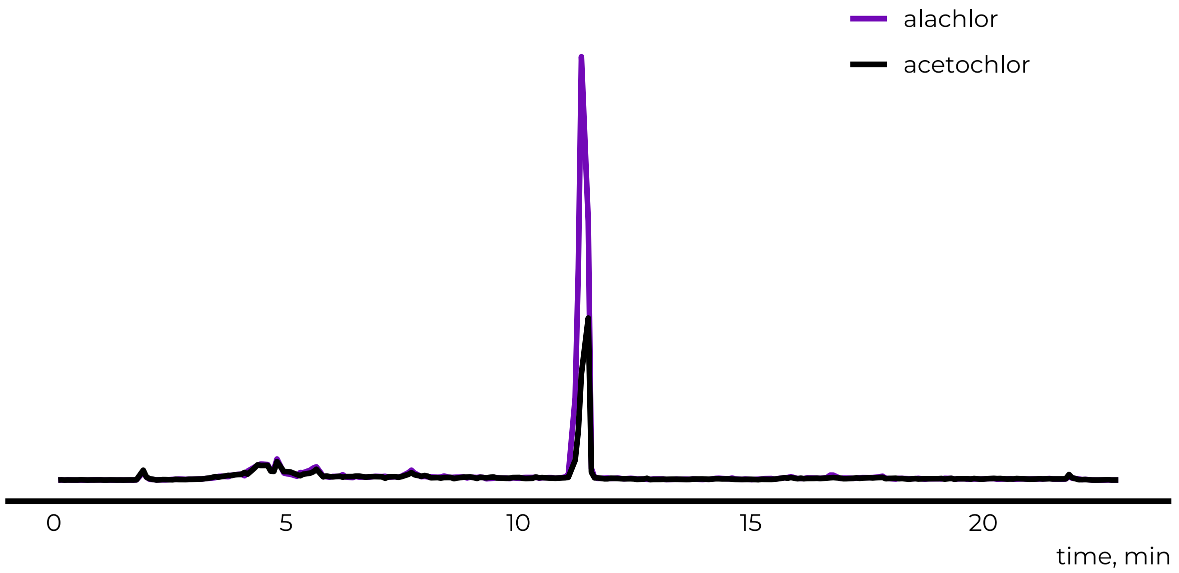

Figure S1 Chromatogram of alachlor and acetochlor.

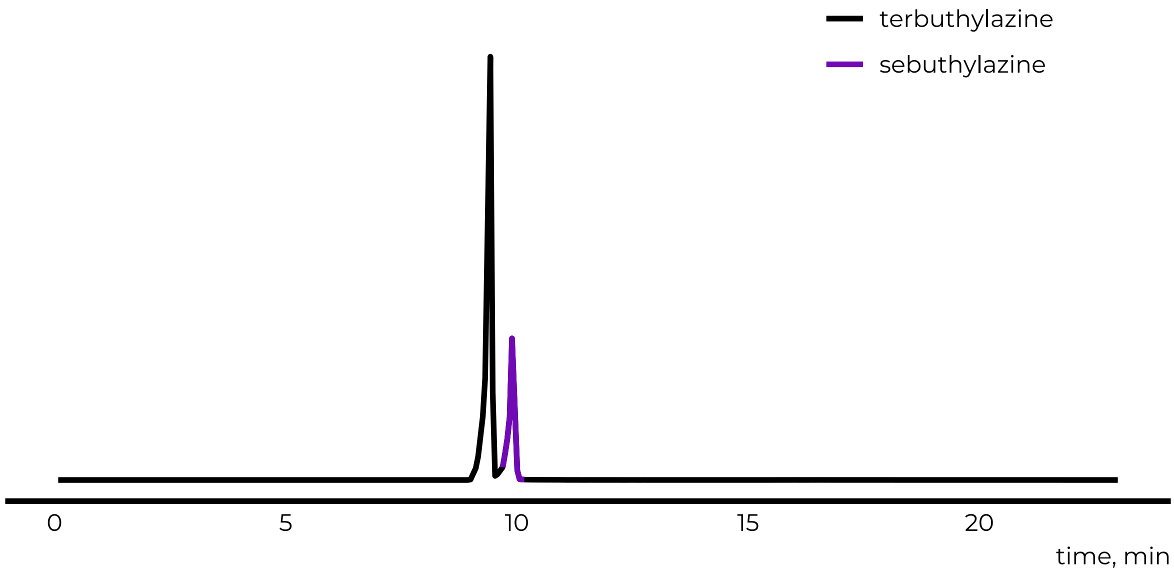

Figure S2 Chromatogram of sebuthylazine and terbuthylazine.

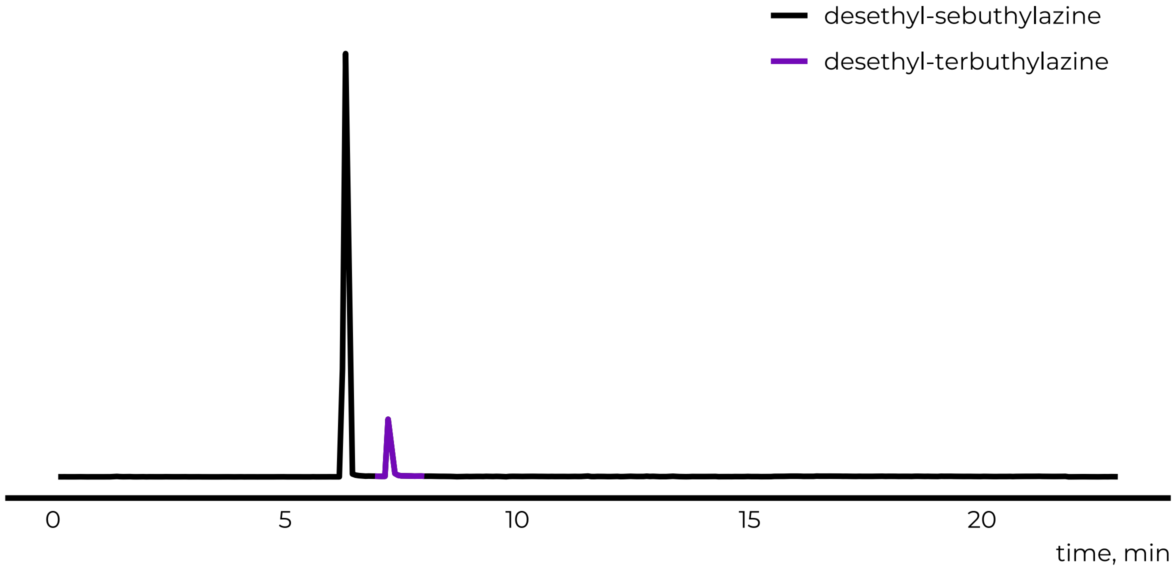

Figure S3 Chromatogram of desethyl-sebuthylazine and desethyl-terbuthylazine.

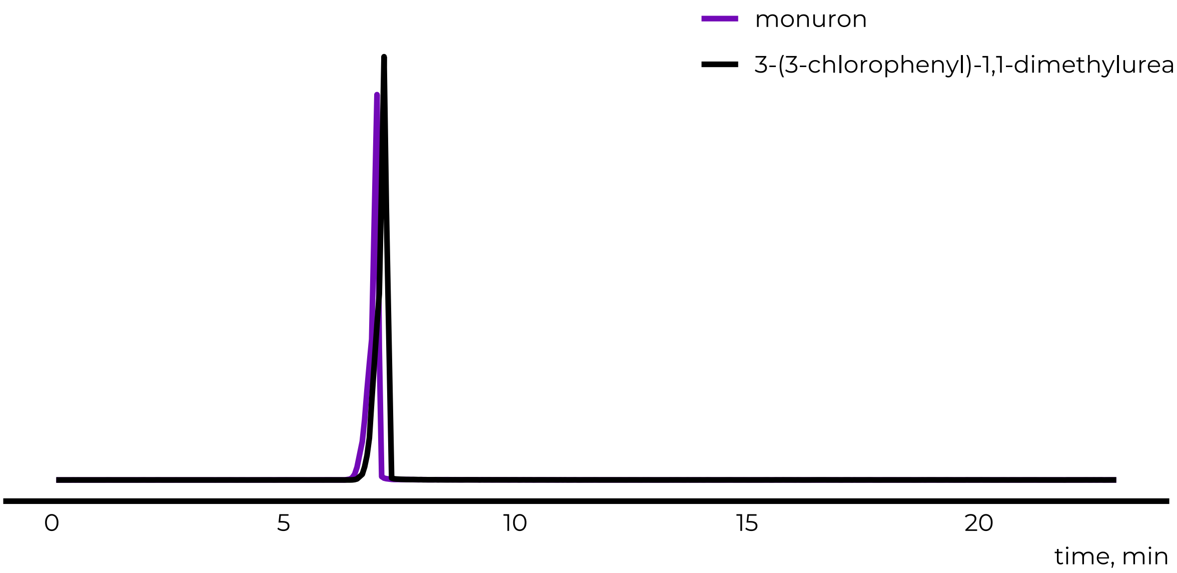

Figure S4 Chromatogram of monuron and 3-(3-chlorophenyl)-1,1-dimethylurea.

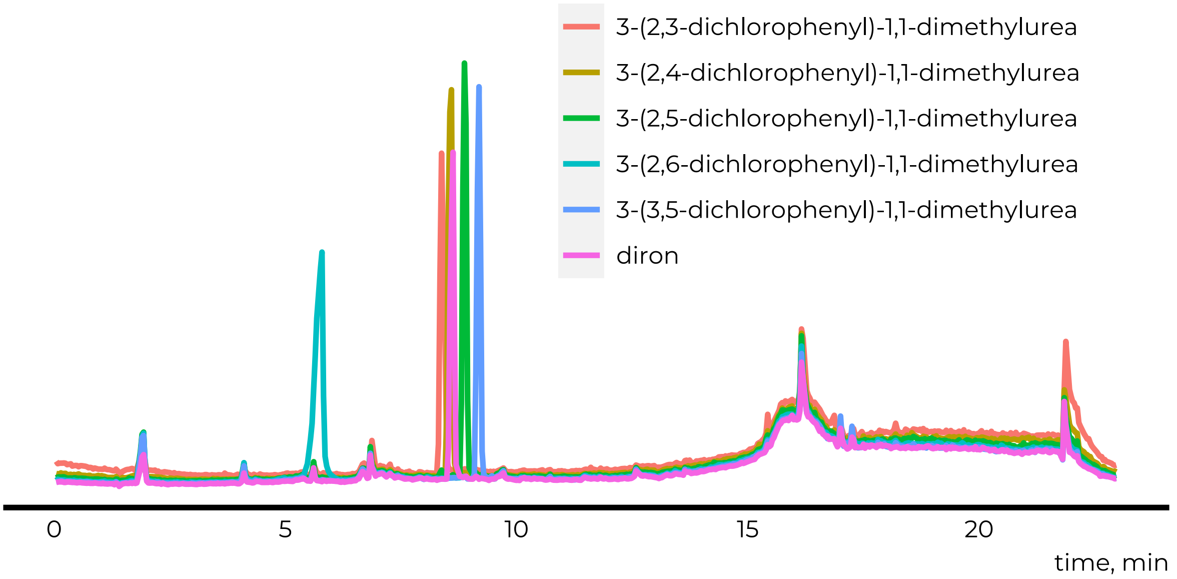

Figure S5 Chromatogram of diuron, 3-(2,3-dichlorophenyl)-1,1-dimethylurea, 3-(2,4-dichlorophenyl)-1,1-dimethylurea, 3-(2,5-dichlorophenyl)-1,1-dimethylurea, 3-(2,6-dichlorophenyl)-1,1-dimethylurea, and 3-(3,5-dichlorophenyl)-1,1-dimethylurea.

Ion mobility separation of the isomeric chemicals

To study the efficiency of high resolution ion mobility to separate the isomeric structure candidates, the ion mobility separation was carried out on a Waters Select Series Cyclic IMS platform with Time of Flight mass analyzer operated in V-mode. Analyses were carried out in direct infusion mode. In electrospray ionization (ESI), the capillary voltage, cone voltage and source offset were adjusted to 1.80 kV, 40 V, and 10 V, respectively. The source temperature was 100 °C, desolvatation temperature was 400 °C, and desolvation gas flow was 600 L/h. Nebuliser gas and reference capillary voltage were held at 6 bar and 1.50 kV each. The Trap CE and Transfer CE were kept at 6 V and 4 V and Post Trap gradient and Post Trap bias were 7 V and 35 V for all measurements. The Driftcell Twave velocity was 375 V and the Twave height was set to 10 V for all ion mobility measurements except multiple cycle experiments where 12 V were used. The cyclic sequence was as follows: 10 ms ‘Inject’, 2 ms ‘Separate’ and 39.6 ms ‘Eject and Acquire’ (allowing for 3 pushes per bin). The separation time was adjusted to achieve the desired number of cycles for each analyte group.

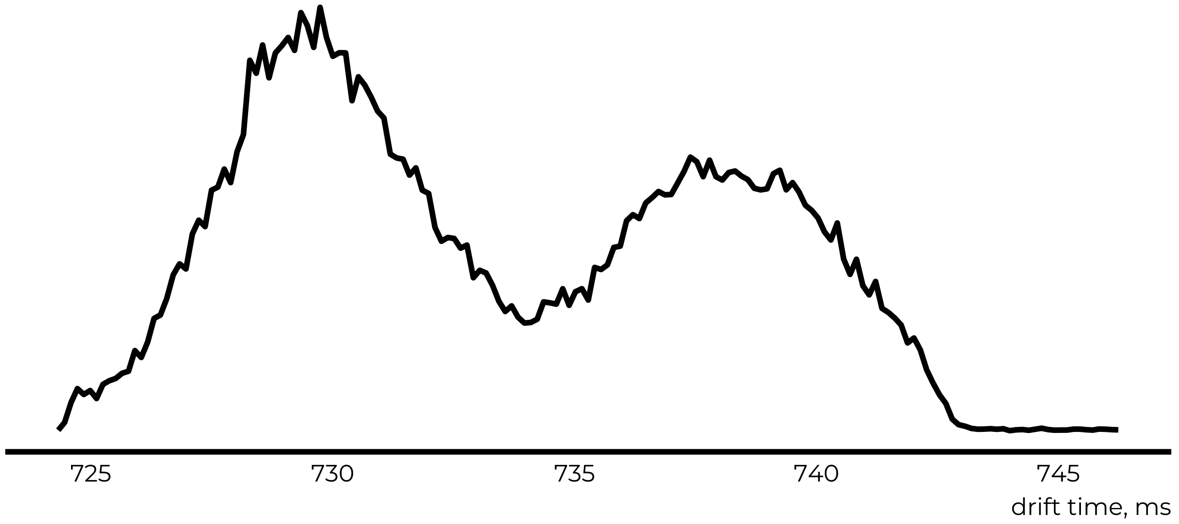

Figure S6 High resolution ion mobility separation (33 cycles) of alachlor and acetochlor.

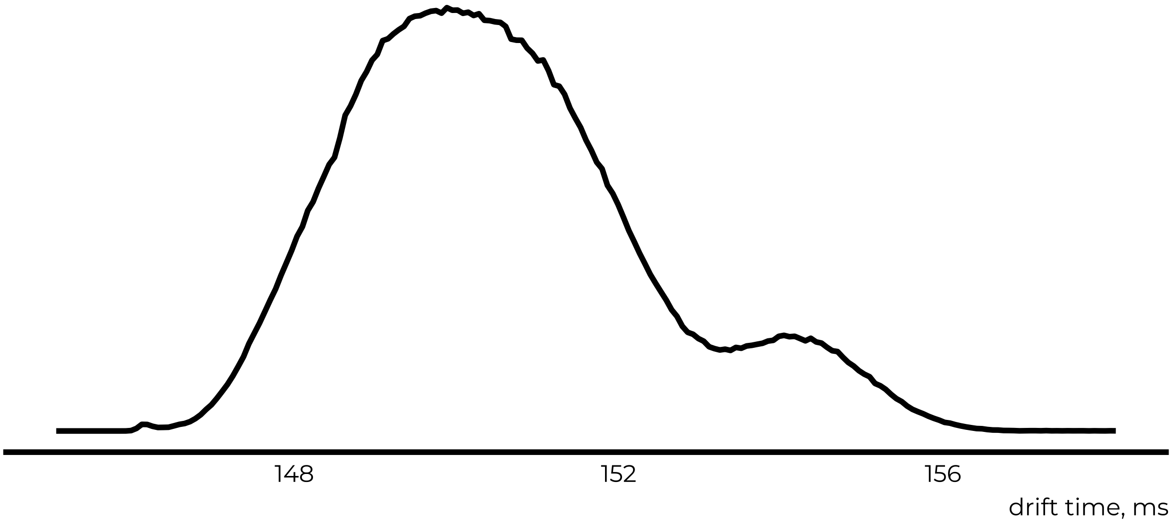

Figure S7 High resolution ion mobility separation (14 cycles) of sebuthylazine and terbuthylazine.

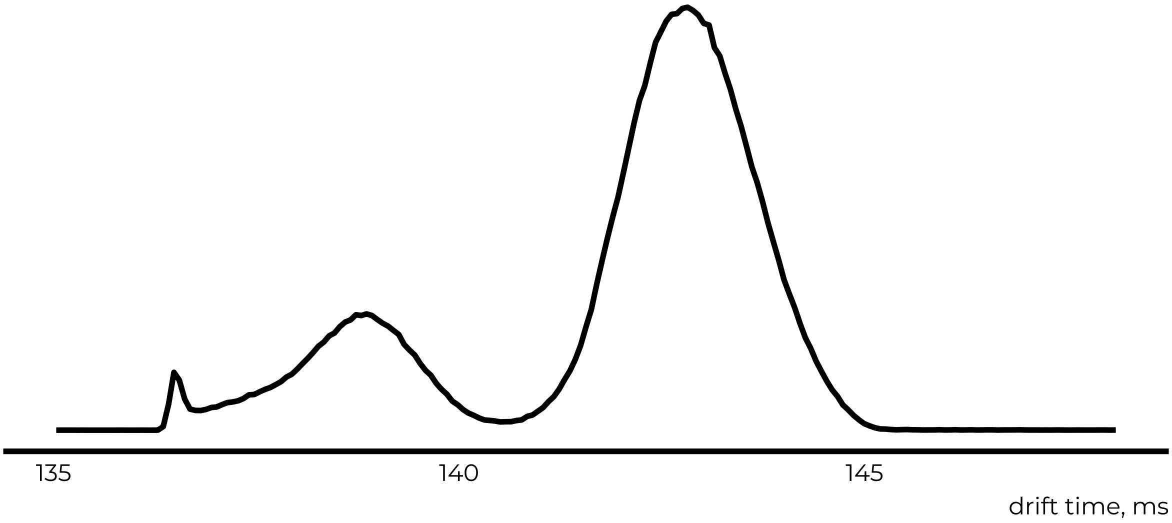

Figure S8 High resolution ion mobility separation (16 cycles) of desethyl-sebuthylazine and desethyl-terbuthylazine.

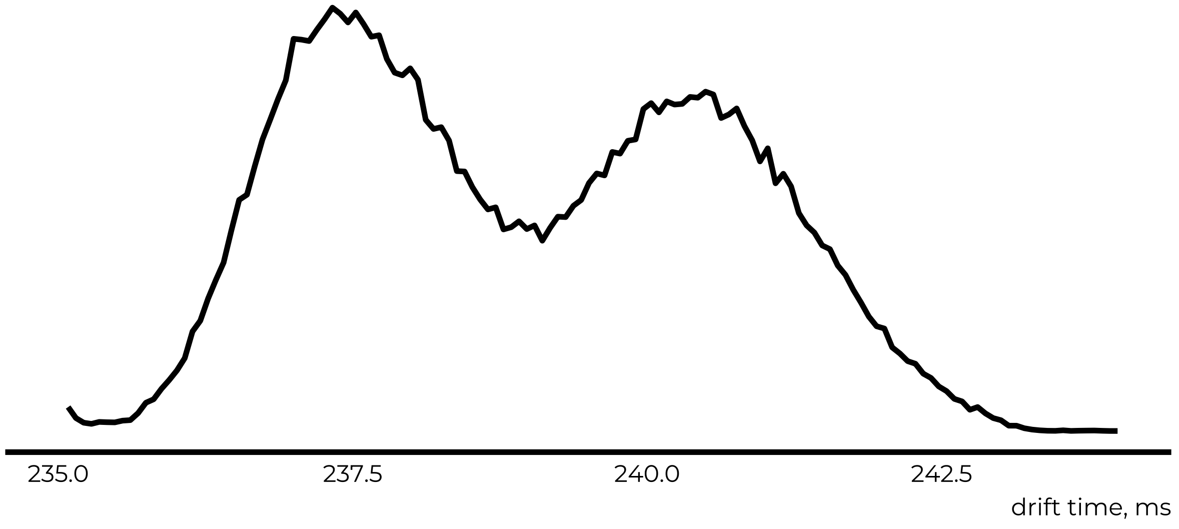

Figure S9 High resolution ion mobility separation (29 cycles) of monuron and 3-(3-chlorophenyl)-1,1-dimethylurea.

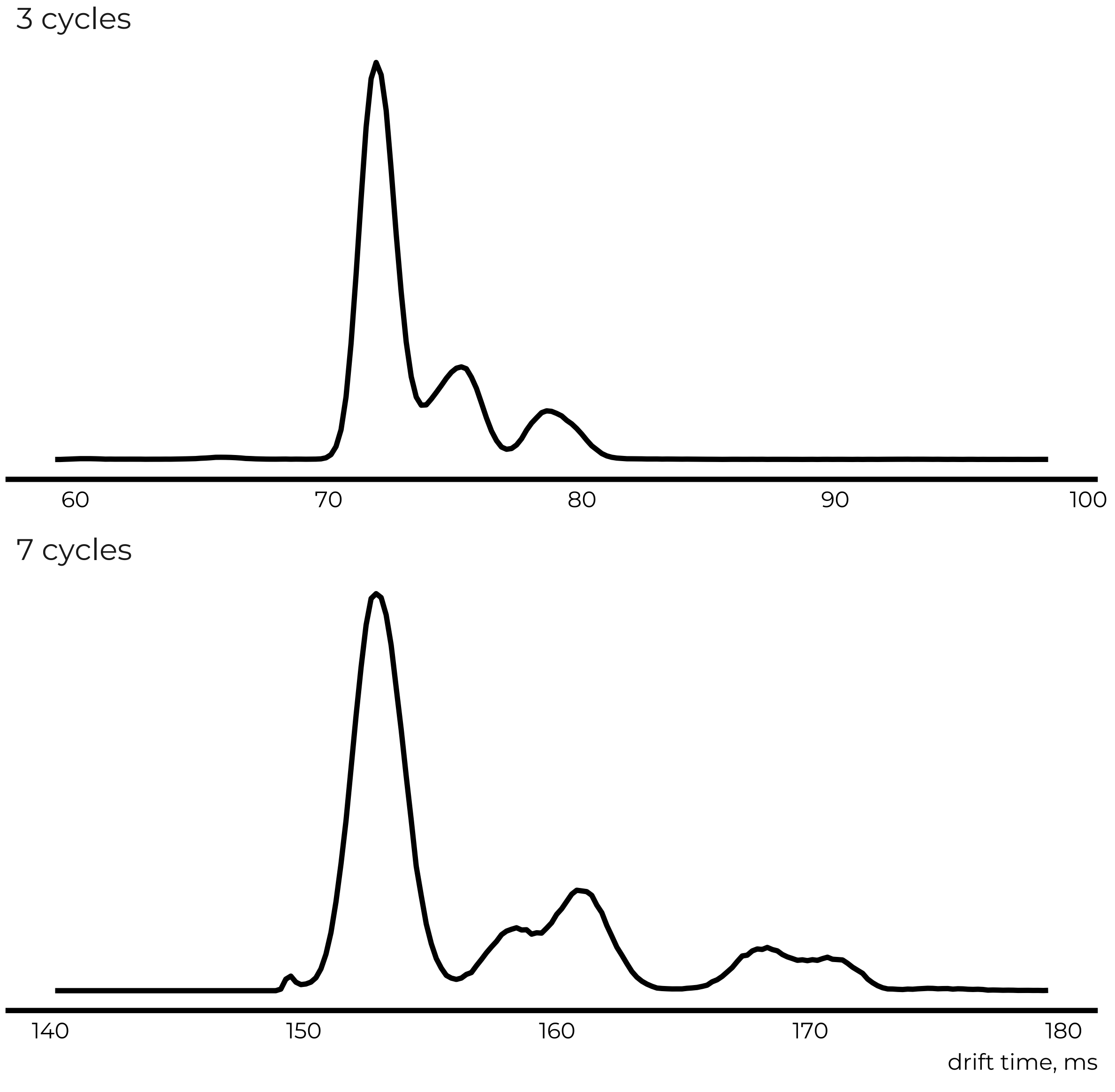

Figure S10 High resolution ion mobility separation (3 and 7 cycles) of diuron and it's five isomers.

MS^2 spectra of the isomeric chemicals

The MS^2 spectra were recorded with direct infusion experiments on Waters Select Series Cyclic IMS platform with Time of Flight mass analyzer.

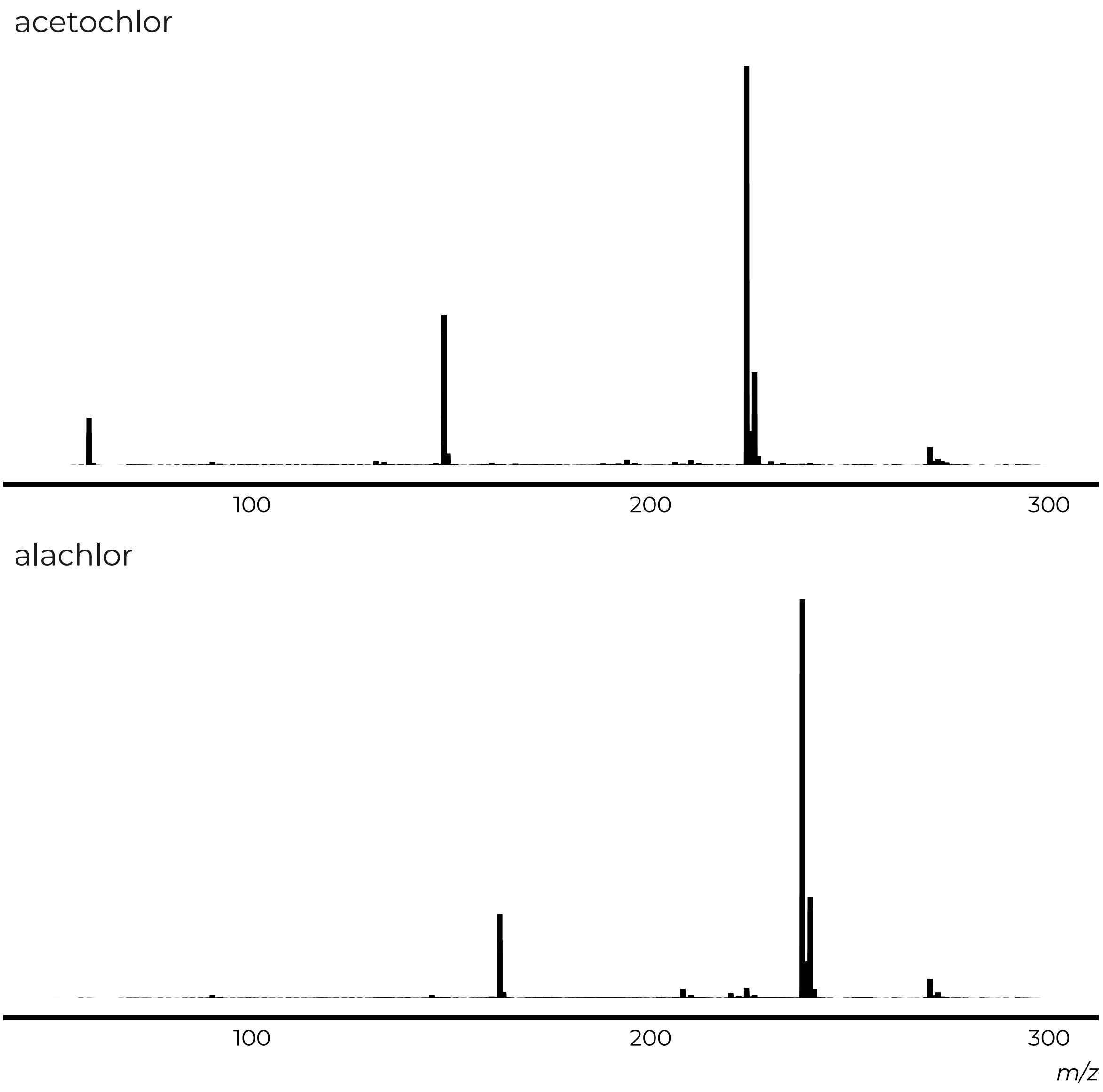

Figure S11 MS^2 spectra of alachlor and acetochlor at 15 V.

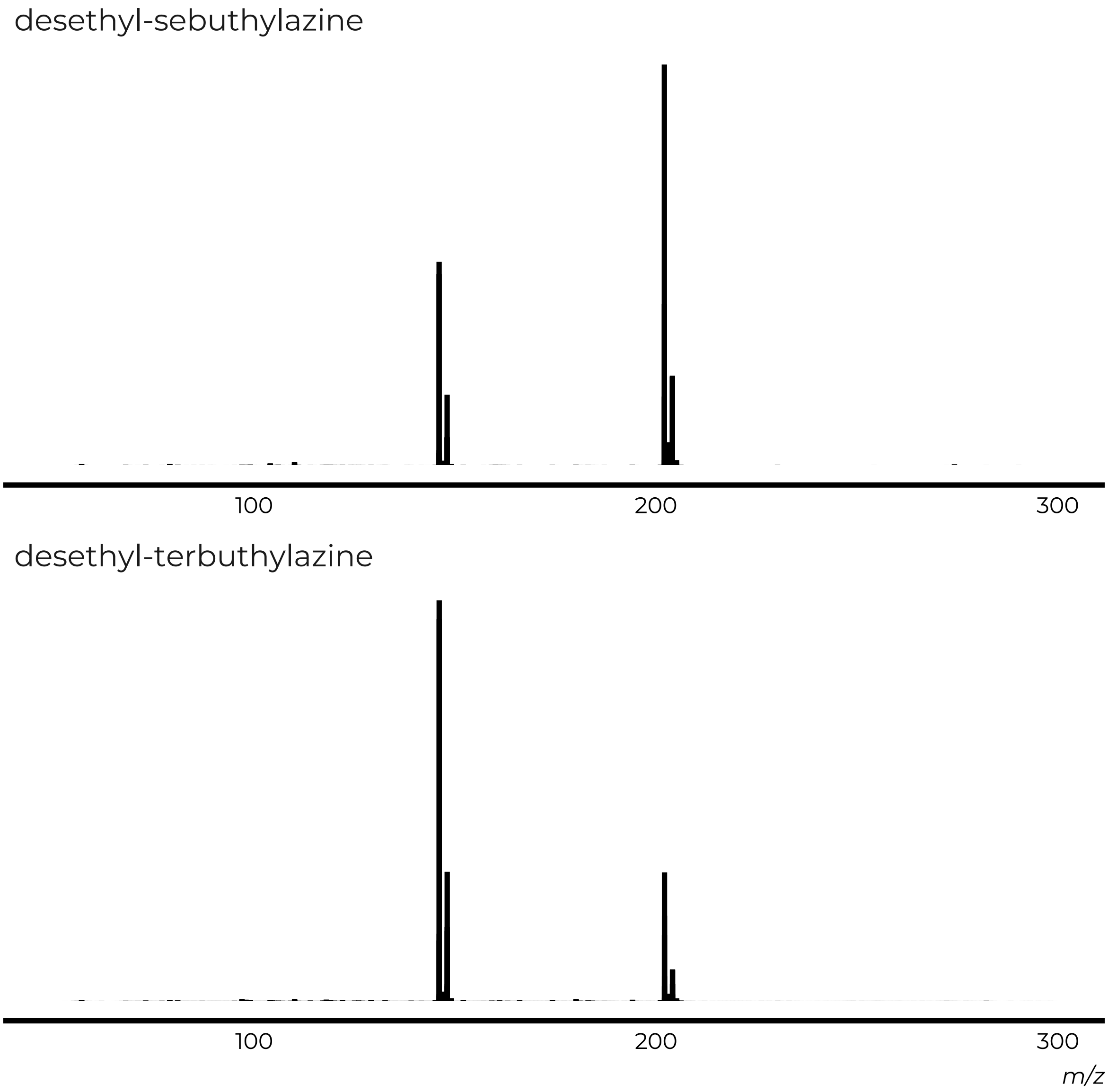

Figure S12 MS^2 spectra of desethyl-sebuthylazine and desethyl-terbuthylazine at 15 V.

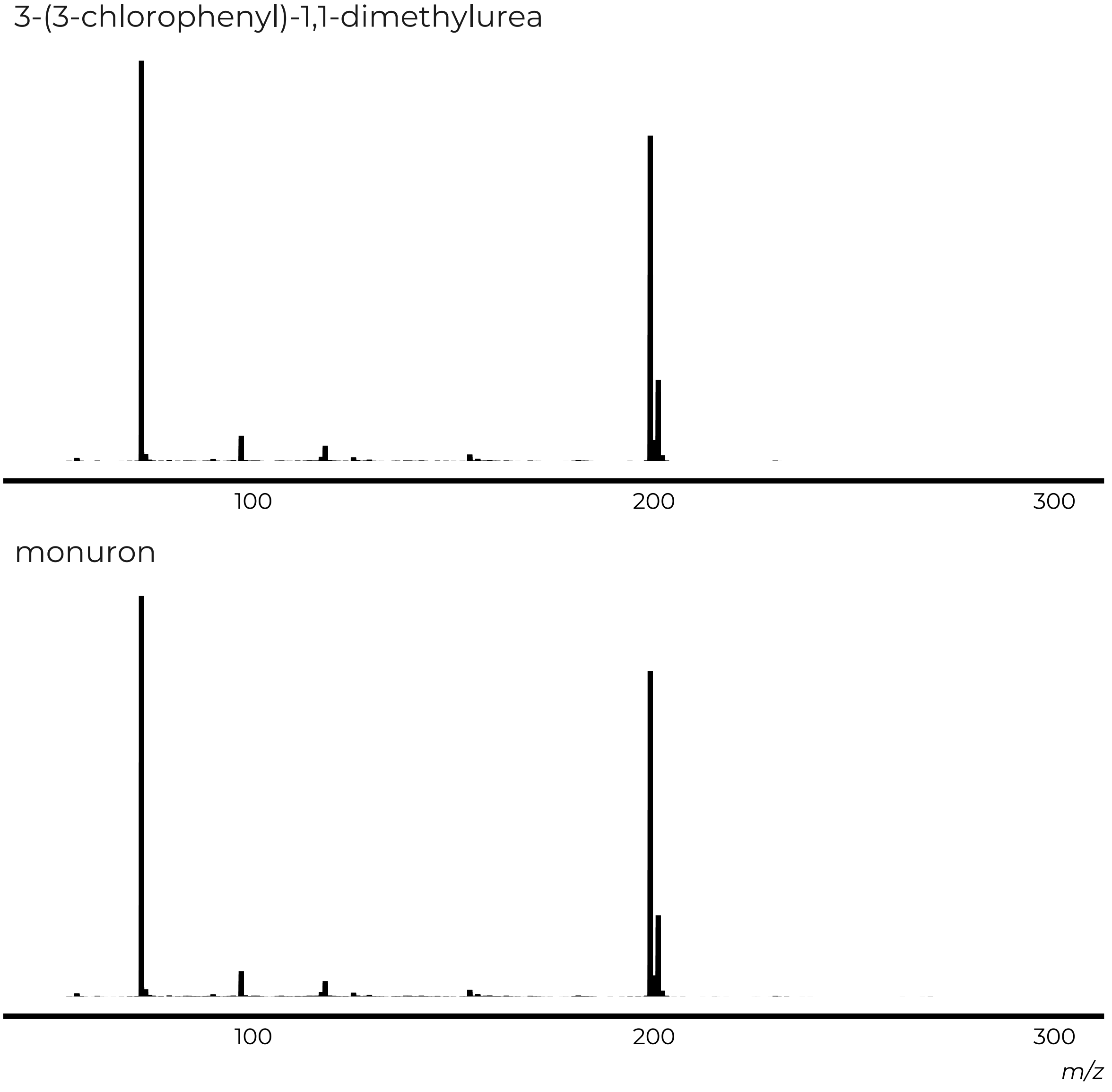

Figure S13 MS^2 spectra of monuron and 3-(3-chlorophenyl)-1,1-dimethylurea at 15 V.

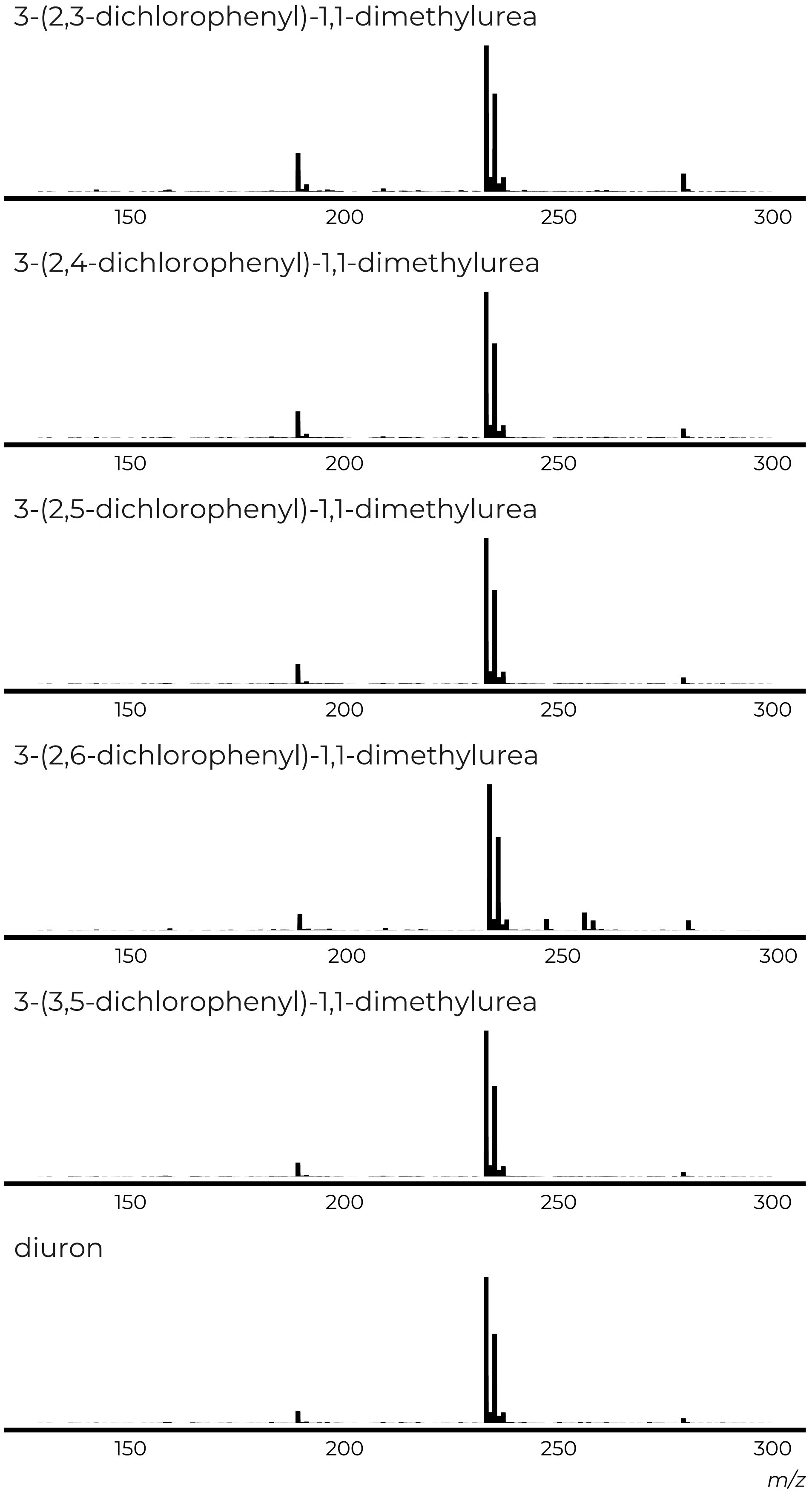

Figure S14 MS^2 spectra of diuron and its five isomers at 20 V.
